# Exploration of shared genetic susceptibility loci between type 1 diabetes and rheumatoid arthritis in the Pakistani population

**DOI:** 10.1186/s13104-019-4590-8

**Published:** 2019-08-27

**Authors:** Muhammad Muaaz Aslam, Peter John, Kang-Hsien Fan, Attya Bhatti, Sidrah Jahangir, Eleanor Feingold, F. Yesim Demirci, M. Ilyas Kamboh

**Affiliations:** 1 Atta-ur-Rahman School of Applied Biosciences (ASAB), National University of Sciences and Technology (NUST), Islamabad, Pakistan; 2 Department of Human Genetics, Graduate School of Public Health (GSPH), University of Pittsburgh, Pittsburgh, PA 15216 USA

**Keywords:** Type 1 diabetes, Rheumatoid arthritis, Association, Shared genetic

## Abstract

**Objective:**

Type 1 diabetes (T1D) and rheumatoid arthritis (RA) are autoimmune diseases. It is known that certain genetic loci and factors that increase the overall autoimmunity risk can be shared among different autoimmune diseases. We sought to replicate seven T1D-related SNPs (single nucleotide polymorphisms) that have been previously reported to be associated with RA susceptibility in a small set of mixed family-based and case–control Pakistani sample in a relatively large and independent RA case–control sample from the same population. Seven T1D-associated SNPs (*GLIS3*/rs7020673, *BACH2*/rs11755527, *SKAP2*/rs7804356, *GDSMB*/rs2290400, *C6orf173*/rs9388489, *LOC399716*/rs947474 and *DLK1*-*MEG2*/rs941576) were genotyped in a large Pakistani RA case–control sample (n = 1959) using TaqMan^®^ SNP genotyping assays.

**Results:**

None of the tested SNPs showed statistically significant association with RA susceptibility; however, one SNP (*GLIS3*/rs7020673) showed a trend for association (OR = 0.88, p = 7.99E−02). Our study has failed to replicate the previously reported association of seven T1D-associated SNPs with RA risk in a large sample from the same population. Thus, our results do not support a major role of these T1D SNPs in affecting RA susceptibility in the Pakistani population.

## Introduction

Prevalence of autoimmune diseases is 3–5% in the general population [1, 2]. Observation of poly-autoimmunity (more than one autoimmune disease in the same person) and/or familial autoimmunity (more than one autoimmune disease in the same family) have suggested that autoimmune diseases share some genetic risk factors and biological pathways [3]. Consistent with this observation, some genetic loci/genes have been found to be associated with multiple autoimmune diseases such as 6p21*/MHC,* 2q33/*CTLA4,* 2q32/*STAT4,* 18p11/*PTPN2,* 1q32/*IL10,* 7q32/*IRF5,* 1p31/*IL23R* [4]. These shared loci/genes may influence the susceptibility for different diseases through the effects of the same or different genetic variations [5–7].

Rheumatoid arthritis (RA) is a complex, systemic, chronic, inflammatory, autoimmune disease characterized by synovial inflammation of mainly small joints and autoantibodies production, which may lead to physical disability associated with the socioeconomic burden and in severe cases, an early death [8, 9]. The interplay of genetic and environmental factors triggers the onset of RA with an estimated heritability of ~ 60% [10]. Multiple genome-wide association studies (GWAS), have identified ~ 50 genetic loci for RA susceptibility [11], with a major contribution from the MHC locus [12].

Type1 diabetes (T1D) is an autoimmune disease characterized by hyperglycemia due to the attack of body’s own immune system on β-cells in the pancreatic islets, reducing the production of insulin which may lead to major health issues such as heart disease, kidney failure, ketoacidosis, blindness and stroke [13, 14]. Contrary to many other autoimmune diseases, which predominantly affect women, T1D affects both genders with a moderately higher prevalence in males at a relatively young age [15]. According to epidemiological data, the incidence of T1D varies globally [16]. To date more than 40 genetic loci have been found to be associated with T1D risk [17].

The concurrence of T1D and RA have been reported within the same individuals and families; however, the shared genetic factors between these two conditions are not fully identified. *HLA*-*DRB1* in the MHC class II region is a major shared genetic locus between these two diseases [18, 19]. Among non-MHC loci, *PTPN22, AFF3, CTLA4, TNFAIP3* and *TAGAP* have been reported as shared risk loci between T1D and RA [20–22]. A previous study conducted in a small set of RA Pakistani sample reported seven T1D-implicated risk variants to be associated with RA risk [23]. In order to replicate or refute this reported association, the present study was conducted in a much larger Pakistani RA case–control sample on the same seven T1D-implicated risk variants.

## Main text

### Materials and methods

#### Study subjects

A total of 1959 unrelated subjects (1222 cases, 737 controls) were recruited for this study. Blood samples and relevant clinical information were collected from two public (Military Hospital and Pakistani Institute of Medical Sciences) and one private (Rehmat Noor Clinic) rheumatology center in Pakistan. All cases recruited in this study (mean age ± SD = 43.1 ± 12.33, 78% women) met the 1987 ACR (American College of Rheumatology) classification criteria for RA [24]. The control subjects (mean age ± SD = 40.7 ± 12.49, 39.5% women) had no history of autoimmune diseases at the time of enrollment. A screening questionnaire was filled out and an informed consent obtained from each subject at the time of the recruitment. All blood samples were collected in EDTA tubes and processed shortly after the collection. The study was approved by the Institutional review board (IRB) of the University of Pittsburgh, USA (IRB no. PRO12110472).

#### Genomic DNA extraction

DNA was extracted from whole blood shortly after collection using either standard phenol–chloroform extraction method or GeneJET Whole Blood Genomic DNA Purification kit (Thermo Scientific USA) and quantified using NanoDrop^™^ 2000 spectrophotometer (Thermo Scientific USA).

#### SNP selection

Seven T1D-associated SNPs which were previously reported to be significantly (p < 0.05) associated with RA in a relatively small Pakistani study sample [23] were selected for genotyping. All the relevant information on the selected SNPs is provided in Table 1.

**Table 1:** Comparison of previously reported and our replication results with RA risk in Pakistanis

Gene/SNP	Chr.	Alleles (minor/major)	Variant type	Previously reported results (Kiani et al. [23])			Our replication results				
				MAF	p-value	FDR	MAF cases	MAF controls	OR	p-value	FDR
GLIS3/rs7020673	9p24.2	C/G	Intron	0.414664	2.86E−04	3.59E−03	0.47691	0.486506	0.9	7.99E−02	2.93E−01
C6orf173/rs9388489	6q22.32	A/G	Intron	0.371429	3.11E−03	1.92E−02	0.370573	0.350714	1.1	1.00E−01	2.93E−01
SKAP2/rs7804356	7p15.2	C/T	Intron	0.195271	2.47E−04	3.59E−03	0.210042	0.218705	0.9	1.25E−01	2.93E−01
MEG3/rs941576	14q32.2	G/A	Intron	0.27612	9.51E−03	4.21E−02	0.323276	0.306525	1.1	2.38E−01	4.16E−01
GDSMB/rs2290400	17q12	C/T	Intron	0.434973	3.48E−04	3.59E−03	0.449324	0.482143	0.9	3.42E−01	4.78E−01
LOC399716/rs947474	10q15.1	G/A	Upstream	0.158732	4.53E−03	2.34E−02	0.199242	0.17617	1.1	4.96E−01	5.79E−01
BACH2/rs11755527	6q15	G/C	Intron	0.404286	9.16E−04	7.09E−03	0.435294	0.421186	1	6.66E−01	6.66E−01

*MAF* minor allele frequency,* FDR* false discovery rate

#### Genotyping

Genotyping of selected SNPs was performed by using available (functionally tested) TaqMan^®^ assays (Applied Biosystems, ThermoFisher Scientific) following the manufacturer’s instructions. After thermal cycling of the TaqMan assays and DNA on 384-well plates, the endpoint fluorescence reading was performed on a QuantStudio™ 12K Flex system (Applied Biosystems, ThermoFisher Scientific). To test the genotyping consistency, 18% of samples were included as the replicates in genotyping runs.

#### Statistical analysis

Minor allele frequency (MAF) for each genotype was calculated based on non-missing allele counts. Congruity to Hardy-Weinberg Equilibrium (HWE) was tested using Chi square goodness of fit test. p < 1E−05 was used to define a significant deviation from HWE as part of the quality control for genotyped SNPs. Logistic regression analysis was performed under the additive model for association analysis of SNPs with RA risk using age and sex as covariates. p < 0.05 was considered as suggestive evidence of association and Benjamin Hochberg false discovery rate (FDR) (q-value) of < 0.20 was considered as statistically significant similarly done in previous studies [25, 26]. All analyses were implemented in R, version 3.4.4. Power analysis was performed using the Quanto Program (http://biostats.usc.edu/Quanto.html).

### Results

A total of 1959 unrelated RA case–control subjects were genotyped to replicate the previously reported association (by Kiani et al. [23]) of seven T1D-associated SNPs with RA. The genotype call rates were more than 90% for all SNPs and the genotype distribution did not deviate significantly from HWE for any of them. All tested SNPs showed similar frequencies in our study as reported by Kiani et al. [23]. Of the seven tested SNPs, only one (rs947474) was an upstream gene variant and the rest were intronic. None of the tested T1D-associated SNPs showed significant association with RA in our large replication sample. However, a trend for association (OR = 0.88; p = 7.99E−02, FDR = 2.93E−01) was observed for one intronic SNP (rs7020673) located in the *GLIS3* gene (Table 1). For the seven SNPs tested in this study with MAFs ranging between 0.16 and 0.43, we had > 80% power to detect significant association at an effect size of ≥ 1.3 under the additive model.

### Discussion

Seven T1D-associated SNPs were previously reported to be associated with RA susceptibility in the Pakistani population using a mixed family-based and unrelated RA case–control sample of 366 subjects [23]. In our study, we genotyped those seven SNPs in a much larger Pakistani sample (1959 unrelated RA cases and controls) and found no significant association with RA susceptibility. However, one SNP (*GLIS3*/rs7020673) showed a trend for the association. This SNP was strongly associated (p = 5.29E−05) with T1D susceptibility in the Pakistani population [27]. In addition to T1D, polymorphisms in *GLIS3* have been reported to be associated with type 2 diabetes, Alzheimer’s disease and osteoarthritis [28, 29]. It is possible that genetic variation in *GLIS3* may also have some role in the RA etiology, but this awaits confirmation in much larger genetic studies.

The difference between the previous Pakistani study [23] and the current study could be related to the differences in the study design and power. The analysis of the previous study was done on a smaller set of combined family-based and unrelated RA cases and controls and that may have inflated the significance level. Our study was sufficiently powered, as we had > 80% power to detect significant associations at an effect size of ≥ 1.3 (> 70% power at an effect size of ≥ 1.25) under the additive model. Alternatively, it is possible that some of those SNPs may be more relevant to the development of familial RA than the sporadic RA since the previous study used a combination of case–control and familial subjects analyzed by FamCC (Family Case Control) software [30].

### Conclusion

In conclusion, our study on a larger RA case–control sample has failed to replicate the previously reported seven T1D-implicated SNPs with RA risk in Pakistanis. The difference in study design, power and contrasting statistical models for data analysis may explain the different outcomes.

## Limitations

Our study could not replicate the previous reported association of T1D-related SNPs with RA in Pakistanis. However, the absence of association should be interpreted cautiously, because the present study is limited by its sample size and sample type. As we specifically explored Pakistani population, the results from this study should not be generalized to other populations. We only tested seven T1D associated SNPs and there is a need to test other T1D-implicated SNPs to explore the shared genetic of RA and T1D.

## Data Availability

The data used to support the findings of this study are included within the article.

## Abbreviations

T1D: type 1 diabetes
RA: rheumatoid arthritis
GWAS: genome-wide association studies
ACR: American College of Rheumatology
IRB: Institutional Review Board
MAF: minor allele frequency
HWE: Hardy–Weinberg Equilibrium
FDR: false discovery rate
FamCC: family case control
SNPs: single nucleotide polymorphisms
